# Therapeutic Evaluation and Utilization Analysis of Mental Health Prescription Digital Therapeutics Within the Current Regulatory Landscape

**DOI:** 10.3390/pharmacy13010019

**Published:** 2025-02-05

**Authors:** Sherry Huinan Xia, Megha Mohan Narayanan, Venkatesh Swamy, Kari Franson

**Affiliations:** Alfred E. Mann School of Pharmacy and Pharmaceutical Sciences, University of Southern California, Los Angeles, CA 90089, USA; huinanxi@usc.edu (S.H.X.); mohannar@usc.edu (M.M.N.); swamyv@usc.edu (V.S.)

**Keywords:** prescription digital therapeutics (PDTs), digital health, mental health, FDA regulations, safety, efficacy

## Abstract

Prescription digital therapeutics (PDTs) are emerging as a pivotal component of digital healthcare, providing software-based therapies for various diseases. This review aims to analyze the regulatory landscape in the U.S., safety, efficacy, and current challenges of PDTs, focusing on mental health conditions. Relevant articles were searched on PubMed, Google Scholar, ClinicalTrials.gov, and FDA Guidance Documents databases, supplemented by manual searches of reference lists from included studies. Inclusion criteria covered English-language studies on the development and application, therapeutic efficacy, and regulatory guidelines of PDTs in mental health. Data extraction and synthesis were conducted to summarize key findings and trends in the literature. FDA regulatory frameworks for PDTs are evolving through pathways of de novo and 510(k) applications, with patient-centric guidance. Clinical trials and real-world data support PDTs’ safety and efficacy, while highlighting regulatory needs. Challenges include payer coverage, patient accessibility, and data privacy concerns. Mixed patient feedback reveals areas for improvement. Limited healthcare provider engagement and payer coverage contributed to financial challenges for PDT manufacturers. Future trends suggest that PDTs will expand beyond mental health. The evolving landscape underscores the need for continued research, regulatory refinement, and collaborative efforts across stakeholders to ensure the successful integration of PDTs into healthcare.

## 1. Introduction

### 1.1. PDT Definition

**Figure 1:**
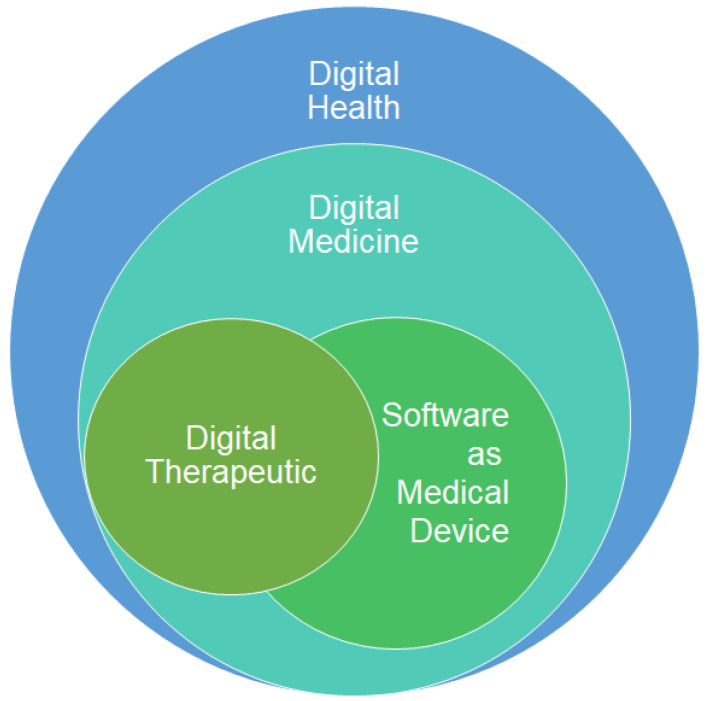
Navigating the PDT landscape.

PDTs represent a rapidly developing domain within the digital healthcare landscape. PDTs are software-based prescription therapies delivered on software platforms that are designed to directly address and manage a broad spectrum of significant diseases and disorders as a subcategory within the domain of digital therapeutics (Figure 1). The devices are accessible via computers, smartphones, mobile applications, wearable devices, or web portals (Figure 1). Their effectiveness has been validated both as stand-alone and combination therapies. PDTs are “cleared” as Class II medical devices based on clinically relevant safety and efficacy data obtained from clinical trials. Typically, FDA-cleared medical devices are not required to undergo clinical trials, unlike FDA-approved products, which must undergo these trials to demonstrate their safety and effectiveness. However, PDTs are an exception according to the FDA regulations that despite being FDA-cleared, they are required to undergo clinical trials.

In contrast to traditional healthcare practices, digital healthcare operates via a patient–machine–HCP interface, providing tailored care and precision medicine through an unconventional workflow [1]. The HCP functions as a consultant, guide, or collaborator, engaging patients actively in the decision-making processes. The health data are disseminated among multiple shareholders, including institutions, hospitals, and patients, facilitated by dynamic point-of-care delivery as long as the patient is present [1].

The objective of this review is to thoroughly examine the therapeutic effectiveness and utilization trends of mental health PDTs in the clinical setting. Additionally, it seeks to explore the impact of PDTs on patient outcomes, the challenges and opportunities in their adoption, and the roles of different stakeholders in facilitating their integration into healthcare systems.

### 1.2. PDTs in Mental Health

The development and access of PDT products is heavily focused on mental health, driven by the convergence of factors including the high burden of mental health disorders, the technological capabilities of personalized medicine, and regulatory support from the FDA through clear pathways. PDTs have emerged as a transformative force in the prevention, management, and treatment of mental health disorders. They provide innovative solutions to address various types of diseases, including MDD, ADHD, PTSD, etc. [2] Mental health PDTs may function as a stand-alone therapy or part of a combination therapy. Stand-alone PDTs in mental health are individual therapies that operate independently of other interventions, providing patients with tailored tools for self-directed mental health management that cater to their individual needs and preferences. Conversely, most mental health PDTs operate as combination therapy concomitant with existing pharmacological interventions as part of comprehensive treatment plans. The integration of PDTs with traditional treatments expands current clinical practices. They offer evidence-based care to reduce patient stigma, improve access to care, mitigate provider shortages, and streamline complex healthcare systems. Additionally, PDTs may lower the risk of adverse events serving as non-pharmacologic interventions.

## 2. Regulatory Pathways

In the U.S., the FDA and other regulatory bodies are currently in the process of developing guidelines for digital therapeutics. Nonetheless, some established guidelines exist to evaluate software in clinical settings, particularly in terms of clinical assessment. The primary oversight of this protocol falls under the IMDRF, a voluntary association comprising international medical regulators [3]. The FDA plays a pivotal role within the IMDRF and holds responsibility for regulating the utilization of PDTs in the U.S. [3].

The FDA regulates PDTs by approving or clearing them as Class II devices [4]. Although PDTs manifest different technology from traditional medical devices, they are reviewed by the CDRH, which may not always align with the iterative and dynamic nature of the software treatments [3].

Following the evaluation of a digital treatment in one or more clinical studies, including randomized controlled trials, the data and formal requests for authorization are subsequently submitted through one of two FDA pathways. Each pathway is characterized by distinct regulatory and evidence-based requirements as outlined below.

### 2.1. De Novo Pathway

The FDA uses the de novo request pathway to classify novel medical devices that demonstrate reasonable assurance of safety and effectiveness but lack a legally marketed predecessor [5]. Devices classified through a de novo request can serve as references for subsequent premarket notification submissions. There are two options for submitting a de novo request for classification into Class I or II [5]. The first option is to submit after receiving a high-level determination of not SE in response to a 510(k) submission [5]. Alternatively, the second option entails submission without preceding 510(k) submission and determining the absence of a legally marketed device for SE determination [5].

### 2.2. 510(K) Pathway

A 510(k) is a submission sent to the FDA prior to the commercialization of a device. Its purpose is to demonstrate the device is safe and effective, establishing it is SE to a legally marketed device [6]. Submitters are required to compare their device to one or more similar legally marketed devices, known as “predicates”, and support their claims of SE [6]. A device is considered SE to a previously approved predicate device if it has the same intended use and technological characteristics [6]. Until the FDA declares a device is SE to a predicate, it cannot be legally marketed in the U.S. Typically, this determination is made within 90 days based on the information provided by the submitter [6]. Upon receiving confirmation of SE, the device can proceed to be marketed in the U.S. [6]. However, if there are changes to an existing device that could significantly affect its safety or effectiveness, or if it is proposed for a new intended use, a new 510(k) submission is required [6].

### 2.3. Graphic Comparison of Regulatory Pathway Timelines (De Novo vs. NDA)

#### 2.3.1. IDE to De Novo Timeline

**Figure 2:**
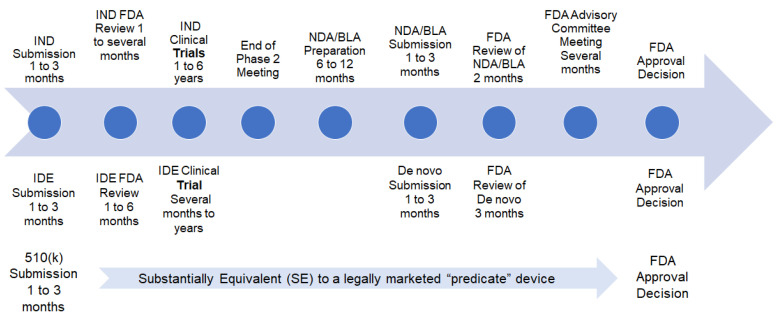
Comparison of drug and device FDA approval timeline.

The process of bringing a medical device to market involves several phases with varying timelines. An IDE is a regulatory submission that allows an investigational device to be used in a clinical study to gather data on its safety and effectiveness. Initiating an IDE submission typically requires one to three months, followed by a 30-day FDA review [7]. Subsequently, the IDE clinical trial phase spans several months to years, allowing for comprehensive evaluation. In cases necessitating a de novo submission, the preparation phase also requires one to three months, followed by a five-month FDA review period [7]. After Phase 2, sponsors can seek guidance on designing large Phase 3 studies, which is crucial before submitting an NDA. The submission of an NDA formally requests FDA consideration for marketing approval, with the FDA having 60 days to decide whether to file it for review. If filed, an FDA review team evaluates the drug’s safety and effectiveness based on the sponsor’s research. The culmination of these phases leads to the FDA’s decision, marking the completion of the regulatory journey for the medical device. The comparison between the approval timeline of IDE and de novo is outlined in Figure 2.

#### 2.3.2. Comparison of NDA and De Novo Timeline

The processes for bringing medical devices and pharmaceuticals to market involve distinct yet complex stages. Submitting an IDE submission for a medical device typically takes one to three months, followed by a subsequent 30-day FDA review [7]. The IDE clinical trial phase spans several months to years. For devices requiring a de novo submission, the preparation phase also takes one to three months, followed by a three-month FDA review [7]. The FDA decision marks the culmination of the regulatory journey. In contrast, drug development entails an IND submission, which takes one to three months for FDA approval [7]. The FDA review process lasts one to several months, and clinical trials span one to six years [7]. Upon completion of Phase II, an End of Phase II Meeting is conducted, followed by a six to twelve-month NDA or BLA preparation, a one to three-month submission phase, and a five-month FDA review [7]. An Advisory Committee Meeting may be held, and the FDA’s final approval decision takes several months [7]. Overall, both processes are intricate and time-intensive, each comprising its unique set of stages and durations (Figure 2).

## 3. FDA Special Considerations for PDTs

User-centered design and human factors are pivotal for PDTs’ success, relying heavily on user engagement. FDA guidelines underscore the importance of incorporating human factor principles into design, including user interfaces, usability testing, and ensuring user-friendliness [8]. Emphasized elements include the provision of clear instructions, effective feedback mechanisms, and flexibility for diverse user needs [8]. As the regulatory landscape evolves, manufacturers are urged to stay abreast of guidelines for successful market entry of innovative digital therapeutics.

Furthermore, PDTs stand apart from conventional pharmaceuticals due to their post-regulatory adaptability, especially when driven by AI or ML technologies [1]. How can a product be approved if it evolves over time and may potentially “hallucinate” responses to input? To address this, the FDA is progressively advancing its comprehensive regulatory framework for digital therapeutics, including PDTs.

Lastly, evolving regulations aim to guide industry players in the nuanced data submissions crucial for establishing safety and efficacy [9]. Instead of evaluating outcomes for clinical and statistical significance, the FDA prioritizes the benefits of PDTs over baseline outcomes in clinical trials, given the assumed lower risk of PDTs.

The FDA’s Digital Health Center of Excellence plays a crucial role in aiding manufacturers through the dynamic regulatory landscape. It serves as a comprehensive resource hub, offering insights into innovation, health equity, cybersecurity, and AI/ML applications in wireless devices. The center has issued 23 guidance documents (in draft and final stages) to assist manufacturers in preparing data for regulatory approval [10].

## 4. PDT Examples with Clinical Trial Data

To gain a comprehensive understanding of PDTs in mental health, we conducted a comprehensive keyword search using databases like PubMed, Google Scholar, ClinicalTrials.gov, and FDA Guidance Documents. The keywords used in our database searches included software as medical device (SaMD), prescription digital therapeutics (PDTs); digital health; mental health. Our research focused on FDA-approved PDTs, covering both de novo and 510(k) pathways, and included all approved mental health and sleep disorder therapies. Key devices examined included reSET^®^, EndeavorRx^®^, NightWare^®^, Sunrise Sleep Disorder Diagnostic Aid^®^, reSET-O^®^, Freespira^®^, Somryst^®^, Rejoyn^TM^, and PRISM^®^ for PTSD. This review provided valuable insights into how these PDTs are innovating patient care and treatment outcomes.

Data extraction and synthesis were conducted to summarize key findings and identify trends over a five-month period starting 15 December 2023 for studies conducted in the U.S. PDTs that received clearance during this timeframe were included. The analysis compared 510(k) and de novo summaries, providing insights into clinical trial data, device functionalities, and the safety and efficacy of these therapies. Patient reviews were also incorporated to assess the effectiveness of the PDTs. ClinicalTrials.gov was a key source for understanding the clinical landscape, and the inclusion criteria centered on the development, application, efficacy, and regulatory guidelines of mental health PDTs.

### 4.1. The First Digital Therapeutic

Abilify MyCite^®^ is an aripiprazole tablet equipped with an ingestible event marker sensor [11]. It is indicated for schizophrenia, bipolar I disorder (monotherapy or with lithium/valproate), and MDD when used alongside other antidepressant medications [11]. Abilify MyCite^®^ tracks medication adherence through a combination of an ingestible sensor, a wearable patch, and a smartphone application. The aripiprazole tablet contains a built-in ingestible sensor made of minerals found in most diets [11]. Upon ingestion, the sensor is activated and emits an electrical signal as it contacts stomach fluid. The signal is then detected by a wearable patch worn by the patient, recording the time of medication intake [11]. The data captured by the patch are subsequently transmitted to a smartphone application via Bluetooth, where medication ingestion data are displayed on a dashboard portal. Authorized HCPs can access the data to monitor adherence.

In 2017, Abilify MyCite^®^ was the first FDA-approved digital medicine system designed to enhance adherence in mental health patients through the Class III (higher risk due to ingestion of the device) premarket approval process [12]. In the trial involving 30 patients with schizophrenia, participants were administered the Abilify MyCite^®^ treatment once daily for eight weeks [13]. The safety sample comprised all 30 patients who used the Abilify MyCite system during the trial. Results indicated that most patients (64.9%) had exposure to the system for 50 to 56 days [13]. Adverse events were observed, with 35.1% experiencing device-associated TEAEs and 32.4% experiencing medication-associated TEAEs [13]. Two serious TEAEs were reported but were deemed unrelated to the treatment [13]. Ten patients discontinued the trial, citing reasons such as adverse events, withdrawal of consent, and protocol deviation [13]. These statistics provide insights into the safety and tolerability of the Abilify MyCite^®^ system in patients with schizophrenia during the trial period [13]. The trial concluded that there were not any safety concerns significant enough to change the labeling of the medication [13].

It raised a controversy despite its approval. The debate around Abilify MyCite^®^ centers on its ability to track medication adherence in patients with mental health conditions. The manufacturer Otsuka claims that such technology can offer valuable insights into adherence patterns, facilitating collaborative efforts between patients and HCPs to address non-adherence issues and improve health outcomes [14]. Notably, Otsuka lacked evidence demonstrating enhanced adherence or improved health outcomes for patients using Abilify MyCite^®^ [14].

Additionally, Abilify MyCite^®^ has raised ethical and privacy concerns regarding the secure handling of sensitive health data. According to Otsuka, the Abilify MyCite^®^ system not only records the timing of medication intake but also tracks the patient’s activity level (number of steps) and resting time automatically [12]. Furthermore, a 2019 study criticized the regulatory approval process, citing insufficient evidence and a lack of improved medication adherence with the digital form of aripiprazole compared to the conventional version [15]. Given the ongoing debate, subsequent mental health software devices utilize various clinically meaningful endpoints to optimize clinical outcomes and diminish healthcare expenditures.

### 4.2. De Novo Devices

Examples of mental health PDTs that have received de novo approval include reSET^®^, EndeavorRx^®^, NightWare^®^, and Sunrise Sleep Disorder Diagnostic Aid^®^. See Table 1 for the summarized clinical trial information.

reSET^®^ (Version 2.0.1): A software application that utilizes a 12-week outpatient augmentation treatment; patients had to currently be enrolled in outpatient treatment for SUD under the supervision of a clinician. It integrated CBT with daily reminders and contingency management (Table 1) [16,17].

EndeavorRx^®^ (Version 2.5.0): A video game that employs sensory stimuli and motor challenges to engage key attention-related brain regions, encouraging multitasking and distraction management through navigation, target collection, and obstacle avoidance tasks for children (Table 1) [18,19].

NightWare^®^ (2024 Version): A software application on the Apple Watch and iPhone platform provided by NightWare, Inc. It detects nightmares in real time using biometric sensors, like the Apple Watch heart monitor. It interrupts nightmares without fully waking the patient using gentle vibrations, improving sleep within two weeks (Table 1) [20].

Sunrise Sleep Disorder Diagnostic Aid^®^: A combination of a sensor placed on the mandible with cloud-based software and a mobile app to analyze sleep data. By tracking mandibular movements, it detects respiratory disturbances, identifies sleep states, and assesses the severity of obstructive sleep apnea, while also providing insights into sleep structure and head position (Table 1) [21]. Healthcare providers can interpret integrated data to diagnose sleep disorders effectively.

The clinical trials for these PDTs have demonstrated both potential benefits and challenges. The benefits include demonstrated effectiveness in targeted populations, enhanced accessibility to treatment, and the potential for personalized interventions. The possible benefits are often lower compared to those of pharmacotherapies. Additionally, several limitations must be addressed, such as the quality and generalizability of the studies, small sample sizes, and the need for further evaluation of long-term safety and efficacy. For example, the clinical trial that led to the FDA approval of reSET^®^ was properly powered and detected significant differences in abstinence rates between the treatment and control groups. In contrast, other products, such as NightWare^®^, faced limitations due to smaller sample sizes. The clinical trial for NightWare^®^ included only 79 patients and was underpowered to detect statistically significant differences in endpoints, primarily because the study was terminated early [20]. Given these challenges, future research should aim for larger, more diverse study populations and extended follow-up periods to gain a more comprehensive understanding of the long-term implications of PDT use.

### 4.3. 510(K) Devices

Examples of mental health PDTs that have been granted 510(k) approval include reSET-O^®^, Freespira^®^, Somryst^®^, Rejoyn^TM^, and PRISM^®^. See Table 2 for the summarized clinical trial information.

reSET-O^®^: A PDT designed for a 12-week (84-day) course, reSET-O^®^ is a software application used alongside outpatient treatment with transmucosal buprenorphine and contingency management to enhance retention in patients with OUD (Table 2) [22].

Freespira^®^: An at-home therapy that teaches patients diagnosed with panic disorder how to regulate and normalize their breathing patterns (Table 2) [23].

Somryst^®^: A digital therapeutic that functions like a personal sleep coach. Using CBT-I, the app guides users through six lessons over a period of up to nine weeks, helping them fall asleep faster and stay asleep longer (Table 2) [24].

PRISM^®^: An FDA-cleared, neuroscience-based prescription therapy for treating PTSD. PRISM^®^ uses a computer simulation and EEG headset to create a non-trauma-based environment where patients can learn to manage their PTSD symptoms effectively (Table 2) [25].

Rejoyn^TM^: A digital therapeutic for treating depression symptoms through brain-training exercises and short, skills-based therapy lessons. Unlike medication, Rejoyn^TM^ leverages the brain’s natural ability to adapt, known as neuroplasticity (Table 2) [26].

### 4.4. Pharmacovigilance

Safety concerns regarding the use of PDTs in real-world scenarios have arisen due to the likelihood of indirect adverse incidents not being immediately recognized during treatment. The negative aspects of technology use are often studied independently from clinical trials that focus on technology outcomes [27]. However, app–app interactions are unpredictable, given the increasing use of portable technology in the largely unregulated digital environment [27]. Therefore, adverse events and unexpected interactions, systemic reports, and meta-analyses should be considered early during the design and development phase of PDTs [27]. In the U.S., the MAUDE database compiles adverse event reports concerning medical devices from manufacturers [28]. It encompasses the latest decade of medical device report data, detailing malfunctions or incidents resulting in death or serious injury. The releasable MAUDE data are accessible to clinicians and the public for voluntary adverse event reporting [28]. However, while the FDA mandates manufacturers to report events leading to death or serious injury, it lacks clear guidance regarding less severe adverse events, and clinicians are requested to use MedWatch form 3500 for voluntary reporting.

In contrast, the Medicines and Healthcare Products Regulatory Agency in the United Kingdom has issued guidance on reportable adverse incidents for digital therapeutics, outlining key considerations for HCPs when evaluating the safety of PDTs [29]. These include performance issues, diagnostic accuracy issues, decision support software resulting in harm, issues with connected hardware or software, human–device interface problems, user error resulting in harm, inadequate labeling or instructions for use, and computer system security problems [29].

#### 4.4.1. Adverse Events

##### Somryst^®^

On 22 May 2022, a MAUDE event was reported involving a patient’s experience of muscular rigidity, anxiety, neck pain, sleep dysfunction, and convulsion/seizure. The nature of the event was classified as an injury [30].

##### EndeavorRx^®^

The reported MAUDE event pertained to a patient presenting no clinical signs, symptoms, or conditions. Categorized as a malfunction, the event description indicated a lack of efficacy [31].

##### NightWare^®^

Two adverse events were reported during the study. Neither was determined to have a probable correlation with the use of the device under investigation. One participant was hospitalized due to a suicidal attempt after study enrollment but prior to the use of the investigational device [20]. Another participant who was enrolled and using the device was diagnosed with sleep apnea, but the contributing factors were determined to be likely present before the device usage (Table 1) [20].

In the context of post-marketing surveillance, a MAUDE event was documented involving a patient experiencing high blood pressure or hypertension [32]. Classified as an injury, the event description specified a medical finding of an elevation in blood pressure, with a noted association with the use of NightWare^®^ [32].

### 4.5. Cost Comparison

In comparison to pharmacotherapy, software medical devices typically recruit fewer study participants in clinical trials, reflecting the unique characteristics of device trials [33]. The availability of alternative treatments may also pose challenges in recruiting voluntary participants. Additionally, conducting medical device clinical trials can be expensive, making smaller sample sizes more feasible from a cost perspective. Consequently, regulatory authorities allow smaller sample sizes for device trials compared to drugs.

Post-regulatory approval, PDT devices generally entail higher upfront costs than drugs for patients. This is attributed to their common adoption of a one-time payment model that covers the length of time that the device was studied, establishing a distinct financial structure [34]. In contrast, pharmaceutical treatments for mental health conditions typically involve continuous duration. Therefore, it is challenging to compare the cost of therapy between devices and pharmaceuticals. These discrepancies must be taken into account when evaluating the cost differences of mental health treatments within the pharmaceutical and software medical device domains.

Currently, there are no cost-effectiveness studies available for PDTs. However, a direct comparison of costs between PDTs and pharmaceutical interventions can be found in Table 3. The average wholesale price of these PDT devices is mostly higher than the first-line pharmaceutical treatments for relevant mental health conditions. Please be aware that Rejoyn^TM^ is not featured in the table as it obtained FDA approval in only March 2024 and is not currently accessible to patients. However, few insurance companies provide coverage for these devices, as such manufacturers may also offer coupons to mitigate costs. For example, Pear Therapeutics, the manufacturer of Somryst^®^, attempted to lower the costs by extending savings cards to eligible patients [35].

## 5. Patient Access to PDTs

The process for a patient to access a PDT involves multiple stages. Initially, a patient consults an HCP to evaluate their mental health condition and determine their suitability for the PDT [39]. The patient will receive a comprehensive education from the HCP regarding the proper use, benefits, and potential risks associated with the PDT [39]. If deemed appropriate, the HCP will then issue a prescription specifying the PDT’s details, typically directly on the product website [39]. Afterwards, the health insurance provider may initiate a prior authorization claim for the prescription [39]. The patient will then acquire the device and/or software with an activation code from a pharmacy, medical supplier, or directly from the manufacturer [39]. Upon obtaining/downloading the PDT, the patient proceeds to set up and activate it according to the instructions [39]. Throughout the treatment, the software application collects data at intervals and generates insights for HCPs to review remotely [39]. Follow-up appointments may be scheduled based on the data collected to assess the treatment’s effectiveness [39]. Adjustments or prescription renewals may be made based on the patient’s response [39]. The overarching goal of the process is to seamlessly integrate PDT into the patient’s healthcare plan, fostering a strong connection between HCP and patient while improving health outcomes [39].

## 6. Real-World Situation

### 6.1. Patient Perceptions

To understand the patient feedback on the mobile application-based PDTs’ usage, we went through the user reviews in the Apple App Store.

reSET^®^: Rated 3.3/5.0 (28 users), with only five users giving the feedback [40]. All the reviews stated that the update made to the application has made the usage worse and unsatisfactory [40]. These reviews mentioned that while the previous version of the application had been instrumental in helping their sobriety and stability, they found themselves unable to utilize the updated version in the same effective manner [40].

reSET-O^®^: Rated 3.2/5.0 (77 users) [41]. The usage showed a slight improvement in user engagement compared to reSET^®^ [41]. However, it still received lower ratings overall [41]. There were mixed reviews about the application interface, its level of engagement, and the effectiveness of its reward-based system [41]. A recurring theme in feedback was dissatisfaction with the rewards program and interface functionality due to the software updates [41].

EndeavorRx^®^: Rated 3.9/5.0 (790 users) [42]. Even though the app received more 5.0 ratings, the common reasons for user dissatisfaction included disinterest in repetitive tasks, frustration with device movement requirements, and disengaging content [42]. Although a few positive reviews highlighted the intriguing software functions and their efficacy in improving conditions, it is notable that the number of positive reviews was quite limited [42]. However, the company has developed a separate mobile application called EndeavorRx Insight^®^ (Companion app) for parents/guardians, where they can monitor their children through the platform, which reflects positive ratings and reviews (rated 4.4/5.0, 136 users) [43].

Somryst^®^: A few articles discussed and compared the existing chronic insomnia treatment with Somryst^®^, highlighting the reduced costs, coverage options, and product availability, but did not provide the patient feedback and prescription fill rate [44,45].

NightWare^®^: One user shared his initial impressions and concerns regarding NightWare^®^, highlighting issues such as the 30 min delay in intervention, lack of documentation, and challenges with the Apple Watch’s performance and technical support [46]. Despite ongoing struggles with nightmares and medication adjustments, he encouraged others to try NightWare^®^ while expressing optimism for potential product improvements [46].

### 6.2. HCP Involvement

The engagement of HCPs plays a vital role to drive the adoption of PDT and potentially improve health outcomes. Initially, HCPs can contribute by streamlining education, support, engagement, and empowerment processes. Through ongoing virtual interactions with patients, HCPs can evaluate the clinical efficacy of PDTs [47]. Secondly, HCPs can offer insights to software developers to better integrate PDT use into their usual workflow within the EHR systems [47]. The integration will achieve security, reliability, and interoperability across entities for the benefit of the patient. Thirdly, HCPs are instrumental in upholding stringent regulatory compliance concerning personal health information data, preserving data privacy and security [47]. Lastly, aligning PDTs with value-based care requires the collaboration of HCPs, who can incorporate these novel technologies into a value-based care framework, thereby optimizing patient care.

## 7. Discussion

Current challenges in the use of PDT include payer dilemmas, limited accessibility to PDT technology, and concerns over patient data privacy. Additionally, despite the comprehensive nature of the review, several limitations should be acknowledged.

### 7.1. Payer Dilemma

In the U.S., the CMS has issued a new code under the HCPCS regarding the use of PDTs for various public and private insurers [48]. In 2022, the American Medical Association Relative Value Scale Update Committee proposed that CMS assign a contractor-priced status (a reimbursement rate negotiated with the contractor/payer) for a new HCPCS code describing digital therapeutics-related care under the Medicare Physician Fee Schedule [49]. As of 2023, only the Massachusetts Medicaid Agency, MassHealth, and Florida’s Agency for Healthcare Administration provide coverage under Medicaid for PDTs [50]. However, the bankruptcy of Pear Therapeutics in 2023 has led the Oklahoma State Medicaid program to take a cautious approach to digital therapeutics due to concerns about their stability and reliability [51].

Contrarily, there is a growing trend of private insurers extending coverage for PDTs via pathways of value-based payment models, flat fee reimbursement, subscription fees, bundled payments, or direct contracts among the plan, employers, and prescribers [52]. However, a dilemma still exists especially among private insurers. While PDTs promise to improve patient outcomes and mitigate healthcare costs in the long run, they often entail significant initial expenses. Payers may be hesitant to cover these devices due to concerns about the upfront financial investment, uncertainty about the devices’ real-world effectiveness, and the absence of well-defined reimbursement mechanisms.

PDTs usually serve as extensions of direct patient care, with a focus on minimizing in-person interactions between HCPs and patients, generating the collection of off-site data for monitoring mental health conditions, and offering real-time data to HCPs. Ultimately, PDTs will enhance behavioral health outcomes for patients [50]. However, payers may face the challenge of determining the value proposition of PDTs. Unlike conventional medications, these devices may not align seamlessly with existing reimbursement models, posing difficulties for payers in assessing their cost-effectiveness and allocating resources appropriately.

Moreover, robust evidence demonstrating the real-world impact and cost-effectiveness of PDTs may be needed, which requires dedicated timeframes and continuous surveillance. The available clinical trials failed to perform “dose”-finding studies to identify optimal usage, recruit diverse participants, reflect significant improvement upon the current standard of care, or explore long-term adverse events [53]. Consequently, payers may be cautious about covering these devices until there is a breakthrough PDTs in which there are sufficient data to support clinical effectiveness.

Therefore, the dilemma for payers revolves around balancing the potential advantages of PDTs in improving patient outcomes and reducing long-term healthcare costs with the immediate financial considerations and the need for robust evidence to justify coverage. Manufacturers are implementing marketing strategies to drive sales of PDTs due to the lack of insurance coverage. For example, Akili, Inc., the manufacturer of EndeavorRx^®^ has introduced EndeavorOTC^®^, a non-prescription alternative for adults with ADHD, utilizing the same technology [18,54]. EndeavorOTC contains mostly identical elements to EndeavorRx^®^ but at a lower cost of USD 24.99 monthly or USD 124.99 annually [55]. While EndeavorRx^®^ prescription targets attention function improvement in children aged 8 to 17 with ADHD, EndeavorOTC^®^ is designed for adults aged 18 years and older. Findings from the STARS-ADHD-Adults trial support EndeavorRx^®^’s effectiveness in adults with primarily inattentive or combined-type ADHD [56]. Freespira^®^ is also switching from prescription to OTC status [57]. The switch offers manufacturers an alternative way of continuous profit, particularly following patent expiration. Also, it can happen only if the manufacturers satisfy the regulatory expectations, such as that the device should be safe and effective for self-use based on clear labeling [58]. Switching a prescription device to OTC might require a new premarket submission to the FDA because directions for safe use by patients differ from those of HCPs. This switch from prescription to OTC might benefit the manufacturer in one way but on the other end. Considering the payers’ perspective, their willingness to pay for it might not be increased. This is mainly due to PDTs becoming one of the alternative options rather than the primary choice of treatment. Until PDT access is expanded and it demonstrates significant benefits for the disease state, payers’ willingness to purchase PDTs is unlikely to change substantially.

### 7.2. Enhancing Accessibility and Integration for PDTs:

The adoption of PDTs by patients and clinicians encounters challenges related to technology accessibility. Older and lower-income adults may lack access to devices compatible with a PDT operating system [59]. Moreover, patients may experience confusion with the rapid implementation of software updates and content enhancements desired by manufacturers [59]. Alternatively, providers confront challenges associated with complex e-prescribing logistics and unfamiliar patient scenarios due to limited training in real-world situations [59].

### 7.3. Addressing Patient Concerns and Privacy

Patient privacy concerns are addressed by HIPAA, ensuring strict protection of EHR. However, specific provisions regarding PDTs are not explicitly outlined [60]. HIPPA Protects the health information of individuals who receive substance use disorder treatment in federally funded programs subjected to additional privacy protections under 42 USC § 290dd-2 and 42 CFR § 2.11 (Part 2) [61]. It provides extra protection related to psychotherapy records compared to normal medical records, most of the notes cannot include any information related to medication prescribed, treatment plan, results, summary of diagnosis, etc. (45 CFR 164.501), and privacy protection requires a covered entity to obtain a patients authorization prior to the disclosure of these notes for any purpose, including any other HCP other than the originator (45 CFR 164.508 (a)(2)) [61]. Some of the policies in place to protect EHR information are inclusion of access control, encrypting the data, and audit trails [62].

Concerns arise regarding patient data protection in situations where manufacturers of PDTs face financial challenges, such as bankruptcy [63]. One such recent example is Pear Therapeutics, which recently sold its product lineup for USD 6.05 million due to financial difficulties, highlighting uncertainties regarding ongoing patient coverage [64].

Ongoing challenges hinder the broad adoption and coverage of PDTs. There is a lack of comprehensive open-source information regarding approved PDT usage, patient perspectives, feedback, and prescription fill rate. Access to such information could benefit stakeholders in better understanding PDT products and addressing existing gaps in knowledge. The evolving regulatory landscape is coupled with rapid advancements in PDT manufacturing, requiring stakeholders, including patients, HCPs, payers, and regulatory bodies, to adapt and integrate these devices effectively into healthcare practices.

### 7.4. Study Limitations

This review has several key limitations. First, there is limited open-source information about PDT usage, patient feedback, and prescription rates, which hinders comprehensive understanding of these products. Second, available clinical trials have methodological constraints, including small sample sizes, lack of diverse participants, insufficient long-term data, and limited studies on optimal usage patterns. Third, the rapidly evolving regulatory landscape and technological advances make it challenging to draw definitive conclusions about long-term outcomes. Finally, financial instability of PDT companies, as evidenced by recent bankruptcies, raises concerns about long-term data accessibility and continued patient support.

## 8. Future Directions

Currently, 9 out of 20 approved PDTs address chronic mental, behavioral, and cognitive disorders, including opioid use disorder, MDD, insomnia, ADHD, etc. [65]. Future PDTs aim to monitor vital signs and address a broader range of therapeutic areas such as blood disorders, circulatory issues, respiratory disorders, and nervous system disorders [66]. The expanding landscape indicates a growing scope for digital therapeutics in diverse healthcare applications. Clinical trial data are typically included in the 510k and de novo summaries as well as other sources, but often remain un-updated on the clinicaltrials.gov website. Investment in PDTs remains limited, which could hold back progress. Without a groundbreaking device that grabs widespread investor interest and sets a clear path for PDT companies, the future might not seem as promising. Some companies are contemplating a shift to OTC products because they believe these can bring in more profits. This transition reflects a strategic move to take advantage of market opportunities and potentially expand revenue sources. However, it also highlights the challenges faced by PDT companies in attracting sufficient investment and establishing a sustainable business model.

The FDA seeks to improve medical device evaluation by collecting post-market, real-world data through the National Evaluation System for Health Technology system, supporting new technology applications, and incorporating real-world evidence in regulatory decision-making guidelines [67].

## 9. Conclusions

PDTs represent a significant advancement in the realm of digital healthcare, offering innovative solutions for the management and treatment of various mental health disorders. Regulatory adaptation and continuous research are essential to ensure safety and efficacy. Despite challenges such as upfront costs, PDTs offer an advanced future and hold promise for a broader healthcare transformation. Continued efforts are needed to harness their full potential and improve patient outcomes across various conditions. Limited investment in PDTs may slow the progress, prompting some companies to consider shifting to OTC status to increase profit opportunities, despite challenges in attracting investors and establishing sustainability. However, collaborative efforts among stakeholders, including HCPs, payers, manufacturers, patients, and regulatory agencies, are essential for achieving meaningful integration of PDTs into treatment plans, demonstrating their effectiveness in real-world settings. In summary, PDTs have the potential to revolutionize the treatment landscape and improve patient outcomes across diverse therapeutic areas. However, further research is needed to address the challenges and embrace the emerging trend in digital therapeutics.

## Figures and Tables

**Table 1:** DeNovo devices summarized information.

PDT DeNovo Device	Intended Population	Study Methods	Safety	Benefits	Patient Perspective
reSET^®^ [16,17]	Patients 18 years of age and older with substance use, opioid use disorder.	reSET was tested in a multi-site, un-blinded, randomized clinical trial to characterize its probable benefits and risks. Study participants received 12 weeks of either treatment as usual (TAU) as standard treatment, or reduced TAU supplemented with a desktop version of reSET (rTAU + reSET).	13% (*n* = 66) patients experienced adverse events: TAU: 11.5% (*n* = 29) reSET + TAU: 14.5% (*n* = 37) No significant difference noted (*p* = 0.3563).	Device exhibited statistically significant improvements in abstinence and retention. Retention rate: 92 out of 252 (36.5%). Dropouts in TAU compared to 71 out of 255 (27.8%) in rTAU with significant difference (*p* = 0.0316).	Satisfaction surveys were conducted on 1–10 scale (1 = very easy, 10 = very difficult). Responses collected from 233 out of 255 participants (91.4%). System and education useful: 8.64/10. Satisfied with the use of computerized system: 8.86/10. Easy to understand: 3.14/10.
EndeavorRx^®^ [18,19]	Children aged eight to seventeen with primarily inattentive or combined-type ADHD and demonstrated attention issues.	EndeavorRx has undergone five clinical studies involving 600+ children with ADHD. In the randomized controlled trial, it enhanced objective attention in eight-to-twelve-year-olds. With sessions lasting ~25 min daily, five days a week for four weeks, recent trials have shown further benefits with an additional month of treatment.	In a clinical trial, 18% of participants experienced mild to moderate device-related adverse events, with the most common being decreased frustration tolerance. No serious device-related events occurred. Three participants discontinued treatment due to decreased frustration tolerance.	Effectiveness results showed significant improvement in ADHD-specific impairment and symptoms for both On Stimulants and No Stimulants groups after one month of treatment. The improvements persisted after a one-month treatment pause and further improved with an additional month of treatment. All improvements had p-values less than 0.001 compared to baseline.	In market research, 90% of caregivers, physicians, and health insurers showed interest in a non-drug digital treatment like EndeavorRx due to its low side-effect profile and attention efficacy. They cited its perceived effectiveness (75%), potential cognitive enhancement (>82%), improved focus (>83%), and ease of use (80%). Additionally, more than 80% believed it could aid in ADHD management. In the ASD pilot study, EndeavorRx significantly improved attention for children, with 73% reporting improvement compared to 50% in the Control group. Parents also noted enhanced real-life attention in 64% of cases. Additionally, 63.6% of parents found EndeavorRx worthwhile, with 90.9% wanting their child to continue using it, compared to lower percentages in the Control group.
NightWare^®^ [20]	Adults 22 years or older who suffered from nightmare disorder or had nightmares from PTSD.	The 30-day, double-blind sham-controlled randomized clinical trial (70 participants enrolled) was performed to study the safety and efficacy of the device. The “sham system” refers to the device consisting of the same components as the active system, but the application only monitored the subjects’ sleep and did not provide any intervention or feedback to the subject.	Patients in both the active and sham arms of the study reported a 1.2-point decrease (less sleepiness) in the Epworth sleepiness scale (ESS) (not statistically significant). Patients in the active arm reported a 0.2-point decrease in the CSSRS, and patients in the sham arm reported no change in the C-SSRS (not statistically significant).	Patients in the active arm had a mean Pittsburgh Sleep Quality Index (PSQI) improvement of 3.2 points, and patients in the sham arm had a mean improvement of 2.2 points (not statistically significant). The secondary outcome of PQSI-A (including sleep disturbance due to anxiety, nervousness, bad dreams, terrors or screaming during sleep) had a mean improvement of 3.3 points in the active arm and 1.4 points in the sham arm (not statistically significant).	Patient perspectives considered for the NightWare Kit (Apple iPhone, Apple Watch, Apple iPhone Charging Cable, Apple Watch Charging Cable) during the review included: Three patients were interviewed on their experiences using the device for 5 weeks. All three patients reported benefit from the device use, noting that the device provided a non-pharmaceutical treatment alternative. The patients noted that the pharmaceutical treatment could leave them feeling “in a fog,” while the NightWare device did not.
Sunrise Sleep Disorder Diagnostic Aid^®^ [21]	Patients 18 years and older with suspicions of sleep breathing disorders.	The sponsor submitted three clinical study protocols and reports to validate the Sunrise SDDA device’s safety and efficacy, focusing on assessing agreement with polysomnography (PSG). Notably, no adverse events, adverse device effects, or deficiencies were reported in these studies, which included diverse patient populations covering different ages, sleep-disordered breathing conditions, body mass indices, and neck circumferences, mirroring the intended U.S. patient demographic. The information utilized in the clinical trial was treated as confidential.	Classified information	Primary effectiveness measured by change in daily sleepiness (time frame: 3 months post-diagnosis), time to diagnosis (time frame: up to 12 months), time to treatment (time frame: up to 15 months), change in daily sleepiness (time frame: 3 months post inclusion). Secondary outcomes were measured by change in quality of life, change in work productivity, cost (€)/QALY, net profit for the French social security system, comparison of CPAP compliance data, comparison of sunrise versus PSG diagnosis, and difference in the obstructive respiratory disturbance index	The FDA document lacks specific information regarding patient perspectives on this device.

**Table 2:** 510(K) devices summarized information.

PDT 510k Device	Treatment	Clinical Trial
ReSET-O^®^ [22]	Treatment for OUD	During a 12-week intervention, the reSET-O + Treatment-as Usual (TAU) group showed a higher retention rate of 82.4% compared to 68.4% in the TAU group alone, a significant difference with a *p*-value of 0.0224. Demographic analysis revealed no significant differences between groups, with most participants being male (54.1%) and white (95.3%), with an average age of 32.9 years. The prevalence of meeting DSM-IV criteria for cocaine dependency was 21.5% in the TAU group and 15.4% in the TAU plus digital therapeutic group, with no statistically significant difference.
Freespira^®^ [23]	Treatment for panic disorder and/or PTSD symptoms. Freespira measures and displays end-tidal carbon dioxide and respiratory rate in real time within a structured breathing protocol	In the single-arm clinical trial (NCT03039231) sponsored by Palo Alto Health Sciences, Inc (*n* = 55; 18 years and older), participants were treated with device for four weeks twice daily via 17 min sessions at home using a sensor and tablet with pre-loaded software. PTSD and symptoms were assessed at the end of the treatment, two months, and six months post-treatment. Primary efficacy outcome: 50% of participants showing ≥six-point decrease in Clinician Administered PTSD Scale (CAPS-5) score at two-month follow-up. Tolerability, safety, usability, adherence, and patient satisfaction were assessed.
Somryst^®^ [24]	For Chronic Insomnia with CBT-1	In a randomized study, participants were divided into two groups: one receiving their standard care along with SHUTi (now called Somryst), and the other receiving only their regular care. The group receiving standard care plus SHUTi showed improvement, as measured by the Insomnia Severity Index (ISI). The average reduction in ISI score was significantly (*p* < 0.0001) greater at week nine and six-month follow-up for the UC+SHUTi arm (mean −7.83 and −8.52 respectively) than the UC+Control arm (means −2.94 and −5.36, respectively). Notably, no adverse effects were reported during the study.
PRISM^®^ [25]	Utilizes EEG signal input for treating patients with PTSD	Gray Matters Health conducted a study to evaluate PRISM as an adjunct therapy for PTSD. It involved 15 EEG neurofeedback sessions over eight weeks in subjects aged 22 to 65 with chronic PTSD, assessing symptom reduction. The study took place internationally, with baseline assessments and pre-training sessions provided. The response rate, i.e., the percentage of subjects (50%) with at least a six-point improvement in Clinician Administered PTSD Scale (CAPS-5) from baseline to the three month follow-up visit (primary effectiveness endpoint) as well as at 8 weeks (exploratory endpoint) was deemed to have been successfully met. While 50.6% (40/79) of the subjects experienced adverse events (AEs), the majority were mild AEs (headache, fatigue) and they recovered right after the training sessions with no further intervention. The pre-specified safety goals of this study were met, and the safety profile was found to be acceptable.
Rejoyn™ [26]	Treatment of MDD symptoms as an adjunct to clinician-managed outpatient care for adult patients with MDD aged 22 years and older who were on antidepressant medication	In a 6-week, multicenter, randomized controlled trial (NCT04770285), 386 participants (aged 22–64) diagnosed with MDD who were on antidepressants were divided into two arms: one receiving Rejoyn, and the other a sham control app. The control app included a cognitive training exercise of shapes memory task. The results indicated that the Rejoyn arm met the primary endpoint by demonstrating a significant mean change on the Montgomery-Åsberg Depression Rating Scale from baseline to Week 6 compared to the sham group (−8.78 vs. −6.66, respectively; with a treatment difference of −2.12 [95% CI, −3.93, −0.32]; *p* = 0.0211). Symptom improvement was also noted through assessment by both patients (Patient Health Questionnaire-9) and clinicians (Clinical Global Impressions-Severity scale). No TEAEs were reported during the trial.

**Table 3:** Cost comparison of PDTs vs. conventional first-line treatment with drugs.

Treatment	Average Wholesale Price (AWP) [36]	Applicable Clinical Trial and Participant Numbers
**Substance Use Disorder (3-month treatment)**		
reSET	USD 2231.91	255
Buprenorphine-Naloxone 8 mg-2 mg Sublingual tablet BID	USD 1963.44	ISTART trial (NCT05362357) and 759
Methadone 10 mg	USD 62.74	OPTIMA trial (NCT03033732) and 272
Naltrexone 50 mg	USD 380.22	NCT02537574 and 380
**Panic Disorder (1-month treatment)**		
Freespira	USD 850–1000	55
Paroxetine 20 mg	USD 76.76	NCT00000368 and 379
**ADHD (1-month treatment)**		
EndeavorRx	USD 621.69	165; 223
Amphetamine-Dextroamphetamine 20 mg	USD 62.41	NCT00507065 and 329
Methylphenidate 20 mg	USD 50.35	NCT01259492 and 725
**PTSD (Continuous treatment)**		
Nightware	USD 7000 (Apple Watch^®^ included)	63
Prazosin 1 mg	USD 46.28 for 30 capsules	NCT00532493 and 304
**Obstructive Sleep Apnea (diagnosis and continuous monitoring)**		
Sunrise Sleep Disorder Diagnostic Aid [37]	USD 399	848 enrolled; still in active state
Positive airway Pressure (PAP) Therapy [38]	CPAP machine rental cost USD 649–USD 989	NCT00051363 and 1105
**Chronic Insomnia (9-week treatment)**		
Somryst	USD 2071.34	N/A
Ramelteon 8 mg	USD 939.26	NCT00237497 and 275
Daridorexant 50 mg	USD 1404.18	NCT03545191 and 930

## Data Availability

All data are contained within the article.

## List of Abbreviations

**Abbreviation**	**Definition**
PDT	Prescription Digital Therapeutics
HCP	Healthcare Provider
SaMD	Software as a Medical Device
MDD	Major Depressive Disorder
ADHD	Attention-Deficit/Hyperactivity Disorder
PTSD	Post-Traumatic Stress Disorder
IMDRF	International Medical Device Regulators Forum
FDA	Food and Drug Administration
CDRH	Center for Devices and Radiological Health
SE	Substantial Equivalence
IDE	Investigational Device Exemption
NDA	New Drug Application
AI	Artificial Intelligence
ML	Machine Learning
TEAE	Treatment-Emergent Adverse Event
CBT	Cognitive Behavioral Therapy
U.S.	United States
MAUDE	Manufacturer and User Facility Device Experience
AWP	Average Wholesale Price
CMS	Centers for Medicare and Medicaid
HCPCS	Healthcare Common Procedural Coding System
OTC	Over-the-Counter
EHR	Electronic Health Record
HIPAA	Health Insurance Portability and Accountability Act
OUD	Opioid Use Disorder
CBT-I	Cognitive Behavioral Therapy for Insomnia
BLA	Biologics License Application
